# Homogenizing Responses to Different Survey Questions on the Same Topic: Proposal of a Scale Homogenization Method Using a Reference Distribution

**DOI:** 10.1007/s11205-013-0335-6

**Published:** 2013-05-24

**Authors:** Tineke de Jonge, Ruut Veenhoven, Lidia Arends

**Affiliations:** 1 Erasmus Happiness Economics Research Organization, Erasmus University Rotterdam, Rotterdam, The Netherlands; 2 North-West University, Potchefstroom, South Africa; 3 Institute of Psychology, Erasmus University Rotterdam, Rotterdam, The Netherlands; 4 Institute of Pedagogical Sciences, Erasmus University Rotterdam, Rotterdam, The Netherlands; 5 Biostatistics, Erasmus MC, Rotterdam, The Netherlands

**Keywords:** Rating scales, Distribution, Scale homogenizing, Rescaling, Meta-analysis, Research synthesis, Happiness, Subjective wellbeing

## Abstract

Survey data are often used for comparison purposes, such as comparisons across nations or comparisons over time. To be effective, this would require equivalent questions and equivalent responses options to the questions. Yet there is a lot of variation in the response scales used, which, for example, differ in the number of response options used and the labeling of these options. This is the case in happiness research, and as a result most of the research data in this field is incomparable. Several methods have been proposed to transform ratings on verbal response scales to a common numerical scale, typically ranging from 0 to 10. In this paper we give an overview of the progress made in those Scale Homogenization methods over time. We describe two early methods: Linear Stretch and the Semantic Judgement of Fixed Word Value Method. Next we discuss the Semantic Judgement of Word Value in Context Method in more detail. Based on these discussions we propose a new Reference Distribution Method. We apply the Semantic Judgement of Word Value in Context and the Reference Distribution Methods to data on happiness in The Netherlands for the years 1989–2009. We show that the Reference Distribution Method produces comparable time series on different questions and that it allows discontinuities in data to be corrected.

## Introduction

Survey research is a major tool of the social sciences and builds on responses to questions using given answer options. There is little uniformity in the questions used and as a result findings on the same topic are often incomparable. This reduces our accumulation of knowledge and calls for techniques to improve comparability of data.

### Diversity in Response Scales and the Comparability Problem

In survey studies, respondents are often given a series of questions with pre-coded response options called ‘response scales’. Different kinds of response scales are used, both verbal response scales and numerical scales and these scales also differ in the number of response options available, some including only two options, for example yes or no, and others as many as 11, for example 0–10 numerical scales.

This diversity in the wordings of questions and in response options also appears in survey research on subjective wellbeing, see for example the large seminal methodological study done by Andrews and Withey (1976), who explored many variations of items^1^ within this theme. The number of items appearing in survey studies has grown rapidly. In the collection ‘Measures of Happiness’ of the World Database of Happiness (WDH) by the beginning of 2011 there were approximately 1,200 items listed (Veenhoven 2011). Though the differences between items are often minimal, this diversity in the measurement of happiness reduces the comparability of the research findings.

One of the aims of happiness researchers is to assess differences in happiness across nations. This requires comparison of data drawn from different surveys containing questions about happiness, but, since the response scales used are often different, only a part of the available research can be used. Likewise, another aim of happiness researchers is to compare happiness within countries over time. This also requires equivalent questions and response scales, but since the response scales can change over the years, the number of comparable data will often be inadequate for a valid comparison to be made.

### Plan of This Paper

In response to the problem sketched above, several methods have been proposed to transform ratings on verbal response scales into a common numerical or continuous scale, typically ranging from 0 to 10. We review these methods in Sects. 3 and 4, and, based on this present a new method in Sect. 5, which we call the Reference Distribution Method. This method can be used to bring different response scales to a truly comparable level on a continuum from 0 to 10. It enables us to extend times series by combining results from different surveys or correcting for discontinuities in trends, and it enlarges the possibilities for comparative studies. We then report a test of this method using survey data on happiness in The Netherlands for the years 1989–2009.

## Diversity in Survey Questions and Approaches to Scale Homogenization

### The Case of Happiness

To provide some guidance for the remainder of this paper we sketch some of the characteristics of response scales used to measure happiness and the results obtained using these measures. This diversity in measures of happiness calls for methods that can be used to transform ratings on different scales into comparable data which over time has led to an ever increasing family of scale homogenization methods (SHM) to be proposed in the literature.

### Survey Questions on Happiness

We define happiness as the subjective enjoyment of one’s life as-a-whole (Veenhoven 1984). In this definition ‘happiness’ is synonymous with ‘life satisfaction’. This concept of happiness is currently the one most commonly used in the social sciences and it lies at the heart of the WDH (Veenhoven 2011).

Happiness in this definition is something that people have in mind and for this reason it can be measured using questioning. The standard question used in the Eurobarometer surveys reads: Taking all together how satisfied are you with the life you lead? Would you say you are: very satisfied, fairly satisfied, not very satisfied or not satisfied at all? There are many variations on this question, some using five response options instead of four and using different verbal labels, such as “extremely satisfied”. The scale used in the Eurobarometer is an unipolar scale: all response options contain the word satisfied. This differs from a bipolar scale, where in the response options, for example, the word dissatisfied would be used to denote the opposite of satisfied.

Next to such questions using verbal response options, there are questions where the responses are rated on a numerical scale. An example is the question on life satisfaction used in the World Values Survey, which reads as follows: Taking all together, how satisfied or dissatisfied are you with your life as a whole these days? Please answer by picking a number between 1 and 10, where 1 stands for “dissatisfied” and 10 for “satisfied”. Variations in numerical scales are seen in the visual orientation, which can be vertical or horizontal and the labeling of the anchor points which can go from negative to positive, for example from −5 to +5; consists only of non-negative numbers starting at 0 or 1; or have no numbering at all (Schwarz et al. 1991; Sangster et al. 2001; Mazaheri and Theuns 2009).

Responses to such questions show that most people are positive about their life, at least in the western world. As a result, the distribution of happiness measurements is skewed, with a long tail on the left that represents ‘negative’ outcomes (Diener and Diener 1996; Cummins 2003). To meet the skewness of the distribution in the past verbal scales have been devised that are skewed due to the mainly positively formulated response options. The idea behind these rather asymmetric scales was that it would give the possibility for more variation in the responses than a more symmetric scale. A scale does not necessarily need to have a neutral midpoint dividing it into a positive and a negative pole, the end points of different scales may also vary in the extremity of the wording used, for example “extraordinarily” is more extreme than “very”, but both terms are subject to the respondents interpretation of the words and this will vary from respondent to respondent. Each of these variations will influence the response patterns (Cummins and Gullone 2000).

Many more variations in survey questions and response scales have been described and studied by Saris and Gallhofer (2007). To date, about 10,000 empirical studies have been done to assess happiness and in these studies some 1,000 different questions have been used all of which can be found in the collection ‘Measures of Happiness’ of the WDH (Veenhoven 2012). The measures are classified by six aspects, see Table 1, and the survey questions presented in this paper are coded according to this classification, see for example Table 2.

**Table 1:** Classification of survey questions on happiness in the World Database of Happiness

Aspect	Example	Code
Keyword used	Satisfaction with life	O-SL
Time reference	Currently	c
Method of assessment	Single question	sq
Kind of rating scale	Verbal	v
Length of rating scale	4-Step	4
Variant of rating scale	Agree–disagree	a, b, …

**Table 2:** Survey items on satisfaction with life used in The Netherlands in two surveys

Item code survey	Question	Response options	Frequencies 2008 (%)
O-SLL-c-sq-v-5-d POLS	To what extent are you satisfied with the life you currently lead?	Extraordinarily satisfied	8.4
		Very satisfied	35.5
		Satisfied	45.1
		Fairly satisfied	7.6
		Not very satisfied	3.4
O-SLL-u-sq-v-4-b Eurobarometer	On the whole how satisfied are you with the life you lead?	Very satisfied	51.5
		Fairly satisfied	44.8
		Not very satisfied	3.1
		Not at all satisfied	0.6

### Overview of Scale Homogenization Methods

The diversity in measures of happiness used, calls for methods to transform ratings on different scales to attain comparable results. In the course of time a number of methods have been developed for this purpose that together comprise a family of what we have named SHM. Each of these methods consists of a way to transform a primary response scale to a common numerical scale and a method to estimate a mean and a standard deviation for this data. We distinguish two methods to estimate a mean and a standard deviation.

- *The Frequency Approach*: this is the common practice where the sample mean is calculated as the weighted sum of the relative frequencies of scores to each of the response options which in turn is used to compute the standard deviation within the sample in the usual way.
- *The* *Continuum Approach*: in this new method the mean and standard deviation of the data are based on the continuous distribution function that best fits the transitions points on a bounded continuum at which verbal response options for a given response scale transit from one to another combined with the frequency distribution of the primary verbal response scale. This approach is described in more detail in Sect. 4.2.

Below, we summarize each of the SHM, in order of progression over the years.

#### Scale Homogenization Using Rank Numbers

In this method the verbal response options of a survey item are subsequently given a rank number, regardless of the semantics of the wordings used to label the options. A mean and standard deviation are obtained by following the Frequency Approach. This method is commonly applied in survey research to analyse the results for items with verbal response options and no transformation is required or considered.

#### Scale Homogenization by Linear Stretch

This is a conventional method by which numerical response options are stretched to a common range from for example 0 to 10, in such a way that the lowest number assigned to a response option is always projected onto 0 and the highest number onto the highest value (10) of the numerical scale and all intermediate options are given equally distanced numbers in between. A mean and standard deviation are obtained following the Frequency Approach. This method is described in more detail in Sect. 3.1.

#### Scale Homogenization by Semantic Judgement of Response Options

This is a group of methods that have in common that experts or judges are deployed to rate the verbal labels of response options on a common numerical scale. We distinguish two variations in this approach.

- *Semantic Judgement of Fixed Word Value*: In this variation experts are asked to rate a series of qualifications that can be given to verbal response options on a common numerical scale. The average rating given to each qualification is kept fixed for every response scale of which it is part. A mean and standard deviation are obtained following the Frequency Approach. The Semantic Judgement of Fixed Word Value Method is described in more detail in Sect. 3.2.
- *Semantic Judgement of Word Value in Context*: In this variation the judges assess the points on a common, bounded continuum at which verbal response options for a given response scale transit from one to another. The Continuum Approach is used to estimate a mean and standard deviation. This variant is described in more detail in Sects. 4.1 and 4.2

#### Scale Homogenization Using a Reference Distribution

The Reference Distribution Method is identical to the Semantic Judgement of Word Value Method in Context except that in the first of these methods the boundaries between the response options of the primary scale are derived from a reference distribution instead of ratings by judges. This new method will be described in detail in Sect. 5.

## Early Scale Homogenization Methods

The Linear Stretch Method is the simplest of all SHM and seems to have been used first by Hull (1922). Other applications can be found in studies on happiness that were performed in Australia and Western Germany in the 1980s (Veenhoven 1993). The study conducted by Jones and Thurstone (1955) and the work done by Lodge (1981) are illustrative of the Semantic Judgement of Fixed Word Value Method. Both this method and Linear Stretch have been used in the WDH since 1990, Linear Stretch to a range from 0 to 10 for numerical scales with at least seven steps and Semantic Judgement of Fixed Word Value for verbal response scales.

### Linear Stretch (SHM–LS)

The Linear Stretch Method is a conventional transformation method and it is best applicable to questions that use a numerical response scale. Scales with five or seven response options are typically stretched to a common range from for example 0 to 10. This is done in such a way that the lowest number assigned to a response option is always projected onto 0 and the highest number onto the highest value of the numerical scale, and all the intermediate options are given equally distanced numbers in between; for example for a 5-point verbal scale the transformation to a 0–10 scale according to this method results in [0.0; 2.5; 5.0; 7.5; 10.0]. The transformed sample mean and standard deviation are obtained following the Frequency Approach. A general form of the formula used to calculate a transformed sample mean based on the Linear Stretch Method can be found in the “Appendix”. When a verbal scale has to be transformed in this way, an initial step is to assign numerical values to verbal response options, typically using consecutive numbers, such as 4 for the most happy option on a 4-step scale and 1 for the least happy option.

Linear Stretch has a number of serious disadvantages. The two most prominent of these are the assumption of equal distances between the response options, and even more problematically, the assumption that the labeling of the response options is irrelevant to the analysis, though not to the respondent. Despite these disadvantages, the Linear Stretch Method is still applied, for example it is used in the WDH for numerical scales with at least seven points to transform them to a comparable scale with a 0–10 range. Another example where the Linear Stretch Method is applied is in the percentage of scale maximum (%SM) method developed by Cummins (1997, 2003). In this method Likert scale data are transformed to a standard form with a range from 0 to 100. In the %SM-method a score of ‘0’ is given to the lowest scale anchor up to ‘n’ to represent the highest scale anchor. Any mean score on this scale can subsequently be converted into %SM units by converting the score into a percentage of the scale maximum value as: %SM = (mean score/n) × 100.

### Semantic Judgement of Fixed Word Value (SHM–SJF)

Over time several attempts have been made to find better methods to cope with the heterogeneity seen in measures on happiness. What many of these alternative methods have in common is that they make use of expert ratings (Veenhoven 1993; Bălţătescu 2002; Lim 2008), that is getting a group of experts to rate the verbal labels of response options on a common numerical scale. An early example of such a method is that of Jones and Thurstone (1955) who requested approximately 900 respondents to rate 51 verbal qualifications on a 9-point Likert scale. A value on a common interval scale and a standard deviation were calculated for each qualification separately. The result was a list of the 51 qualifications ordered on the bases of their value on the common interval scale.

A Semantic Judgement of Fixed Word Value Method is also applied in the WDH, Veenhoven (1993) and 12 co-workers rated the degree of happiness denoted by the verbal labels of 29 commonly used survey items on a numerical 0–10 scale. For example, the label “very happy” was an option in 8 of the 29 items and it was given a rating varying from 9.2 to 9.4 resulting in an overall mean of 9.3, whereas an overall mean of 3.7 was found for the label “not very happy”. This method is still used to transform responses reported in the WDH for scales where using the Linear Stretch Method is deemed inadequate or incorrect. The transformed sample mean based on the Semantic Judgement of Fixed Word Value Method can be calculated in a manner similar to that used for the Linear Stretch Method. The formula used to do this can be found in the “Appendix”.

The Semantic Judgement of Fixed Word Value Method as applied in the WDH overcomes the disadvantages of the presumed equidistance of the response options and the neglect of labels associated with the Linear Stretch Method: however, the Semantic Judgement of Fixed Word Value Method also has some weak points. Kalmijn (2010) mentions that the fixed values applied in the WDH

- are based on expert judgements that do not necessarily reflect the views of non-expert respondents,
- have been rated by Dutch experts on basis of the English version of the questions, thus implicitly assuming that the feelings associated with an item are not affected by its translation from Dutch into English,
- do not take into account the phrasing of the lead question, nor the number and the labels of the alternative response options and their position on the scale.

## Later Scale Homogenization Method Using Semantic Judgement of Word Value in Context (SHM–SJC)

The weaknesses of these early transformation methods also appeared when the transformed scores were compared to average ratings on 0–10 numerical scales in the same country in the same year (Kalmijn et al. 2011). These weaknesses instigated two further innovations.

### Innovation One: The Happiness Scale Interval Study

In order to counter the shortcomings of the Semantic Judgement of Fixed Word Value Method, Veenhoven (2008) started the Happiness Scale Interval Study. This study was set up to look at survey questions on happiness using verbal response options, such as “very happy” and “pretty happy” with the intent to determine consistently what degrees of happiness are denoted by such terms when based in different questions and languages. These degrees are expressed in numerical values on a continuum ranging from 0 to 10. The main purpose is to identify the numerical values at which respondents change their judgement from for example “very happy” to “fairly happy” or the reverse. Identification of this point is obtained by asking experts to rate the turning point from one to another response option on a continuum of 0–10 using a web-based Scale Interval Recorder (Veenhoven and Hermus 2006).

#### Technique of the ‘Scale Interval Recorder’

**Fig. 1:**
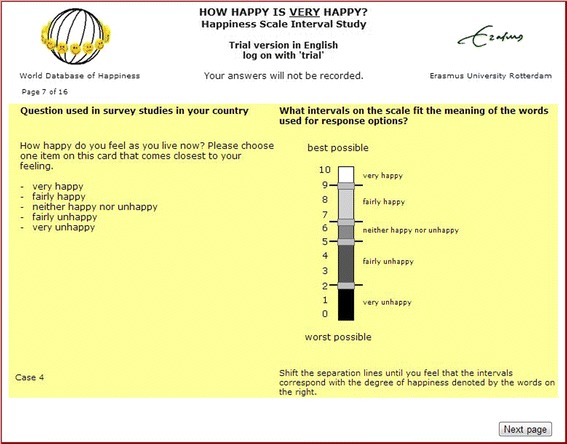
Screen shot of the Scale Interval Recorder

In this method a series of survey items is presented on a computer screen to what are referred to as ‘judges’. Items are presented sequentially on the left side of the screen and each item presented consists of a question and corresponding verbal response scale with options given in the judges’ mother tongue. An example of the Scale Interval Recorder is given in Fig. 1. On the right side of the screen a vertical bar scale is displayed with a number of small horizontal slides on it, the number of which is equal to the number of response options minus one. The judges have to shift the slides until they feel that the intervals on the vertical bar correspond to the meaning of the words as used for the verbal response options. Note, the response options that are displayed next to the bar move simultaneously with the slides to the level of the mid interval value of each interval.

Looking at Fig. 1 it can be seen that the extremes of the numerical bar scale are labeled “Worst possible” and “Best possible”. In the terminology of Saris and Gallhofer (2007) these labels are called ‘fixed reference points’. What worst and what best means, is left to the interpretation of the judges. The labeling of the extremes is thus semi-abstract which makes them applicable to all questions presented to the judges and independent of the subject of an individual question. An additional advantage of this semi-abstract labeling is that the judgement is not influenced by the extremity of the wordings used for the labels of the end points of the continuum.

#### Difference with Early Methods for Scale Transformation

The approach to scale transformation used in the Happiness Scale Interval Study differs essentially from that used in the Linear Stretch Method and the Semantic Judgement of Fixed Word Value Method, as the response options in the primary scale are not considered to be discrete points, but to be intervals each representing a part of the continuum from 0 to 10 where the perception of happiness can be found. This complies with the view of Kalmijn (2010), who considers happiness to be a latent continuous variable that underlies the survey questions being studied. Moreover, in the Happiness Scale Interval Study each response option is judged in the context for the other response options of the scale and this approach is illustrative of the Semantic Judgment of Word Value in Context Method.

#### Empirical Illustration

To illustrate how the three methods are used we selected two survey items fielded in the Netherlands. The first was taken from a survey named Permanent Onderzoek Leefsituatie^2^ (POLS) of Statistics Netherlands and the second from the Eurobarometer. The POLS-item has an asymmetric response scale with five options. The Eurobarometer item has a symmetric response scale without a neutral midpoint and four options. The items are summarized in Table 2 which also includes the frequency distributions for this data for 2008.

The labels of the response options will not be interpreted in the same way by all respondents. Some people may consider the labels of all the response options of the POLS scale to be positively formulated, whereas others may interpret the two options at the lower part of this scale as negative expressions of satisfaction with life. Some people may believe one cannot be less satisfied than ‘not at all satisfied’ and will consider this option to be the null point of the Eurobarometer scale, while others may believe things can be worse and assign an interval of positive length to this option. Interpretation of semantic intervals will vary from person to person for all kinds of reasons such as personality, cultural context or the context of the scale (Hazelrigg and Hardy 2000). As a consequence, in the Happiness Scale Interval Study items are assessed by a group of judges. This results in a report of the average value and the variance for each boundary between two response options. This implies that the results should be considered as representative for the population and are not applicable for subgroups with specific characteristics.

**Fig. 2:**
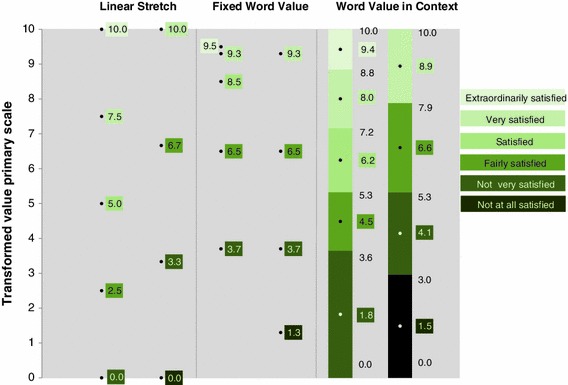
Comparison of transformations using three methods: Linear Stretch, Semantic Judgement of Fixed Word Value and Semantic Judgement of Word Value in Context

The two items presented in Table 2 together comprise six response options, three of which are included in both items. The transformation of the response scales of the items to a scale from 0 to 10 according to each of the three transformation methods is depicted in Fig. 2.

From Fig. 2 it can be seen that in the Linear Stretch Method the extremes of both primary scales are pinned to 0 and 10 and that all the other response options are equally spaced in between. When the Linear Stretch Method is applied the response option “fairly satisfied” of the 5-point scale is assigned the transformed value 2.5, whereas this option for the 4-point scale gets a transformed value of 6.7. This large difference between the values 2.5 and 6.7 is elucidatory for the fact that the wordings of the response options are neglected when Linear Stretch is applied.

If the Semantic Judgement of Fixed Word Value Method is applied the results are entirely different. The value of a label such as “fairly satisfied” is fixed in this method and equal to 6.5 according to the Dutch experts, however, from Fig. 2 it can also be revealed that the Semantic Judgement of Fixed Word Value Method treats each response option as isolated from the other options and thus does not take into account the context of the scale.

As can be seen in the Semantic Judgement of Word Value in Context Method the assumption of equal distances between response options is abandoned and the idea is promoted that a fixed value applies to a label of a response option, irrespective of the labeling of the other options. If we consider the response option “fairly satisfied” once more, we can see that this option is assigned the interval 3.6–5.3 for the 5-point scale, with a mid interval value equal to 4.5 and a length of 1.7. For the 4-point scale the interval for this option ranges from 5.3 to 7.9, with a mid interval value of 6.6 and a length of 2.6.

At the start of the Happiness Scale Interval Study, the Frequency Approach was applied to the Semantic Judgement of Word Value in Context Method results and used to estimate a mean and a standard deviation, analogues to how this is done in the Linear Stretch Method and the Semantic Judgement Fixed Word Value Method. A comparison of the results obtained using these three SHM is shown in Table 3.

**Table 3:** Transformed means obtained using different transformation methods (frequencies 2008)

Item code survey	Linear stretch	Semantic judgement of fixed word value	Semantic judgement of word value in context
O-SLL-c-sq-v-5-d POLS	5.9	8.6	6.9
O-SLL-u-sq-v-4-b Eurobarometer	8.2	7.8	7.7

The survey items from POLS and the Eurobarometer address more or less the same topic and mainly differ in the response scales. The results for each item are assumed to be representative for the Dutch population and therefore one would expect that given that a transformation method is applied, the transformed means for 2008 would be equal. This is clearly not the case. The difference of 2.3 between the transformed means of 5.9 and 8.2 based on the Linear Stretch Method is most striking.

### Innovation Two: The Continuum Approach Applied to Semantic Judgements

The calculation of a transformed sample mean based on mid interval values still treats happiness as a discretely distributed variable, just as the two older methods. This does not do justice to Kalmijn’s view that happiness is a latent continuous variable of nature. To deal with this Kalmijn introduced the Continuum Approach as an appropriate alternative for calculating a sample mean on a continuum from 0 to 10 (Kalmijn 2010). He proposed the beta distribution as an appropriate distribution for this approach in relation to the measurement of happiness, which is defined by two positive shape parameters, *α* and *β* and can expressed using the complete beta function.

1 $$ B(\alpha ,\beta ) : = \int\limits_{0}^{1} {t^{\alpha - 1} (1 - t)^{\beta - 1} dt} $$

Given the formula (Eq. 1) the probability density function of the beta distribution on the continuum from 0 to 10 can be written as:

2 $$ f(x|\alpha ,\beta ) := \left\{ {\begin{array}{*{20}c} {[10B(\alpha ,\beta )]^{ - 1} x^{\alpha - 1} (10 - x)^{\beta - 1} } \hfill & {{\text{for }}\,x \in [0,10]} \hfill \\ 0 \hfill & {\text{otherwise}} \hfill \\ \end{array} } \right. $$

**Fig. 3:**
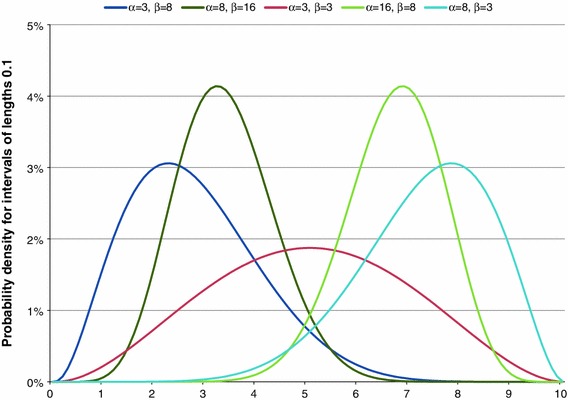
Examples of the probability density functions of the beta distribution

To make this less abstract we give some examples of the probability density functions of the beta distribution for different values of *α* and *β* in Fig. 3.

From Fig. 3 it can be seen that if *α* and *β* are reversed, the shape of the probability density function is mirrored vertically. If *α* is smaller than *β*, the function is skewed to the right, if *α* is larger than *β* the function is skewed to the left and if both parameters are equal the function is symmetric. Furthermore, the larger the values of *α* and *β*, the steeper and narrower the density curve is. The estimates for the parameters *α* and *β* can be used directly to estimate the transformed sample mean μ as:

3 $$ \hat{\mu } = \frac{{\hat{\alpha }}}{{\hat{\alpha } + \hat{\beta }}} $$

In the joined Semantic Judgement of Word Value in Context Method and the Continuum Approach the boundaries obtained from the Happiness Scale Interval Study are combined with the associated frequency distribution to estimate the parameters of the best fitting beta distribution. There is always a perfect fit for a response scale with three response options. If the number of response options is restricted to only two, then the situation is undetermined and the number of possible beta distributions is infinite. If the number of response options is at least equal to four, then in general there will be no perfectly fitting beta distribution and the best fitting solution should be taken. Those who are interested in the methodological considerations of this approach can find more information about it in Kalmijn (2010, Ch. VI) and Kalmijn et al. (2011).

**Fig. 4:**
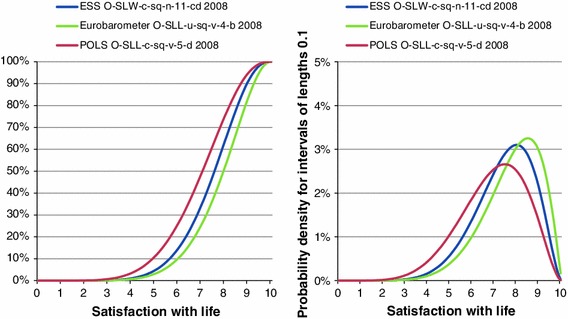
Distribution of happiness in the Netherlands in 2008: estimates using the Semantic Judgement of Word Value in Context

The two verbal scales shown in Table 3 are convenient to demonstrate the scale homogenization process when applying the innovated Semantic Judgement of Word Value in Context Method. Before doing this however, we will introduce another scale to serve as a reference to evaluate the results of the transformations. This reference was taken from the European Social Survey (ESS), which contains the question: All things considered, how satisfied are you with your life as a whole nowadays? The answer has to be rated on an 11-point numerical scale from 0 to 10 with the extremes labeled “extremely unsatisfied” and “extremely satisfied”. Just as for verbal response scales, a best fitting beta distribution can also be estimated for discrete numerical scales. In the Semantic Judgement of Word Value in Context Method this is at present done by assuming that all ratings of the primary scale represent equally wide subintervals on a 0 to 10 continuum (Kalmijn 2013). The beta distribution based on this ESS-scale was chosen as a reference, since this numerical scale, although probably not perfect, comes closest to the continuum from 0 to 10. The transformation results for the three scales using the best fitting beta distributions are depicted in Fig. 4. The left graph shows the cumulative distribution function, the density function is shown on the right.

As stressed before, since the results for all three transformed scales were based on survey responses made in 2008 to similar items, one would expect the three curves to more or less coincide. This is obviously not the case. Compared to the reference distribution, the distribution for the Eurobarometer item is too skewed to the left and that for the POLS item too skewed to the right. For the Eurobarometer item this can be explained by the fact that the primary scale offers the response options “fairly satisfied” and “very satisfied”. Respondents who are satisfied with their life thus have to choose between an option that either underestimates or overestimates their perception of satisfaction with life. Apparently a majority of the satisfied respondents tend to prefer the option “very satisfied” over the option “fairly satisfied”, which pushes the beta distribution to the right. The explanation for the POLS item lies in the strong asymmetry of the primary scale in which four of the five options are formulated positively. As a consequence the option “satisfied” in the primary verbal scale is positioned in the middle of the scale, which may not be in accordance with the position a satisfied respondent would expect its position to be. Furthermore, as can be seen from Fig. 2, the judges valued the position of the option “satisfied” in this context rather low on the continuum. As a consequence, the beta distribution for the POLS item falls to the left of the reference distribution.

The estimated population means according to the different methods are presented in Table 4.

**Table 4:** Transformed means for different transformation methods (frequencies 2008)

Item code	Linear stretch	Fixed word value	Word value in context (frequency approach)	Word value in context (continuum approach)
O-SLW-c-sq-n-11-cd ESS	7.7	–	7.5	7.4
O-SLL-c-sq-v-5-d POLS	5.9	8.6	6.9	6.9
O-SLL-u-sq-v-4-b Eurobarometer	8.2	7.8	7.7	7.7

The Semantic Judgement of Fixed Word Value Method does not allow the calculation of a transformed mean for the ESS item, since the latter has only labeled extremes, however, based on the discussion of the construction of the primary scales of the POLS item, we can conclude that a transformed mean of 8.6 is far too high to be realistic. We would not expect the mean to be substantially higher than the transformed mean for the Eurobarometer item.

Of all methods the means obtained using the joined Semantic Judgement of Word Value in Context Method and the Continuum Approach come closest to the transformed mean for the reference item, yet they still leave a large gap in between the transformed means of this reference item and are far from identical. We have also noticed these differences in outcomes for other survey items, although these showed smaller deviations of the transformed means to that of the reference item than is the case for the items taken from the Eurobarometer and POLS surveys. In the remainder of this paper when we talk about the Semantic Judgement of Word Value in Context Method we imply it is combined with the Continuum Approach.

Since the results for the items taken from the Eurobarometer and POLS surveys were the worst compared to other items we looked at, these two items were chosen as illustrative examples to show that an additional step has to be added to the Semantic Judgement of Word Value in Context Method to solve the comparability problem. Nevertheless we could conclude that the Semantic Judgement of Word Value in Context Method in general shows a smoother pattern of results than either the Linear Stretch Method or the Semantic Judgement of Fixed Word Value Method. The Semantic Judgement of Word Value in Context Method alleviates many of the shortcomings of the two older methods. Moreover in contrast to the older methods, the Semantic Judgement of Word Value in Context Method does do justice to the continuous nature of the latent variables that underlie the survey questions being studied.

## A New Scale Homogenization Method Using a Reference Distribution (SHM–RD)

The observed differences in transformed distribution means between items discussed above for all SHM inspired the first author of this paper to devise a method in which a reference distribution is used to ‘tune’ responses to other questions on the same topic across surveys.

### Deriving Boundaries from a Reference Distribution

The Reference Distribution Method for making happiness data comparable builds heavily on the Semantic Judgement of Word Value in Context Method. Basically the two methods are identical except that in the Reference Distribution Method the boundaries between the response options of the primary scale are derived from a reference distribution instead of from ratings by judges on a Scale Interval Recorder.

With the Reference Distribution Method an attempt is made to deal with the fact that, for a given year and a given population, one would expect the transformed distribution means for similar questions about happiness asked in different representative surveys to be approximately the same irrespective of the primary response scales used: yet as we have shown in the preceding sections, this is not the case when using the methods described in Sects. 3 and 4. We have explained that this is a by-product of the fact that the verbal scales used in for example the Eurobarometer and POLS items do not necessarily offer response options that meet the perception of respondents well, which forces them to choose between two less than optimal alternatives. The least inappropriate option may be ranked in a counterintuitive position in between the other response options. As a consequence, the boundaries derived from the assessments made by native language speaking judges may not correspond with how the response options are selected in practice by respondents.

To find a solution to this problem a different angle of approach is needed (Dijkgraaf 2008). Instead of taking verbal scales that have to be transformed as the point of departure, the beta distribution that fits best to the survey results of a deliberately chosen item in a given year is used as the reference distribution to start the transformation of other scales. Preferably, this reference distribution is based on survey results measured on a continuum from 0 to 10. In general survey results measured on a continuous scale will not be available. As a second best solution a representative survey item with a numerical scale should be selected and used to estimate the best fitting beta distribution that can serve as the reference distribution. If however, only verbal scales are available for a type of item that all consist of a similar question but vary in scale, one of these items has to be selected as a basis for the reference beta distribution. The Scale Interval Recorder can be deployed to obtain the values of the boundaries between the response options for this selected item. Combined with the frequency distribution for the selected item in a reference year the parameters of the best fitting beta distribution can be estimated and used as the reference distribution.

Once a reference distribution is available, its cumulative distribution function can be used to derive the boundaries between the response options on a continuum from 0 to 10 for any other survey item addressing a similar question, but with a different scale, that has been fielded in the same year as the reference distribution. These boundaries follow straightforwardly from the cumulative distribution of the reference distribution and the cumulative frequencies for the response options in the primary scale: the boundary between response option i and response option i + 1 is equal to the point on a continuum from 0 to 10 where the value of the cumulative distribution of the reference distribution is equal to the sum of the frequencies corresponding to the response options 1 up to and including i in the primary scale.

**Fig. 5:**
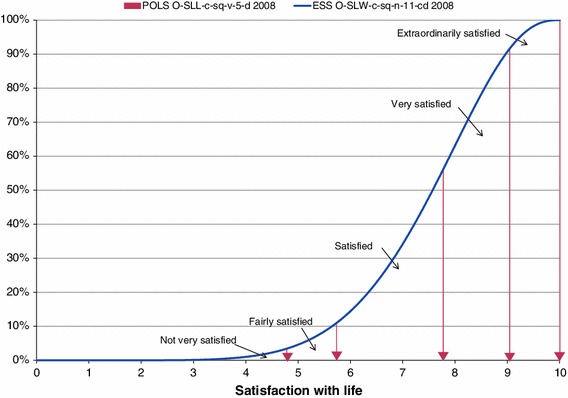
Illustration of the Reference Distribution Method to derive boundaries between verbal response options

How boundaries in the Reference Distribution Method can be derived from a reference distribution is shown in Fig. 5. The beta distribution based on the survey results for the ESS item introduced in Sect. 4.2 is used here as a reference to derive the boundaries between the response options of the scale of the POLS item taken from the survey results for 2008.

In Table 2 a frequency of 3.4 % was denoted as the response to the option “not very satisfied”. In the cumulative reference distribution this percentage is reached at the value 4.8 on a continuum from 0 to 10. Of all respondents, 7.6 % selected the option “fairly satisfied”. Together with the 3.4 % for the response option “not very satisfied”, this adds up to 11 %. This percentage corresponds to the value 5.7 on a 0–10 continuum, which is the upper boundary of the interval for the response option “fairly satisfied”. Continuing this way, upper boundaries of 7.8, 9.0 and 10.0 can be found for the options “satisfied”, “very satisfied” and “extraordinarily satisfied”. Then using these boundaries and the frequency distribution for the POLS item as measured in 2008, the parameters of the best fitting beta distribution can be estimated. As might have been expected, this best fitting beta distribution coincides with that found for the ESS item we presented in Fig. 5.

**Fig. 6:**
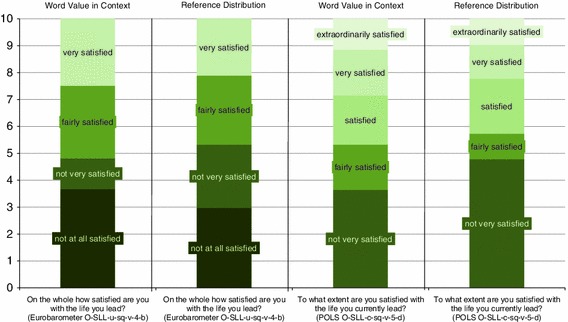
Boundaries as based on the assessments by judges or based on a reference distribution

An obvious question of interest is how the boundaries found using the Reference Distribution Method relate to the boundaries obtained using the Semantic Judgement of Word Value in Context Method, where the boundaries are based on assessments made by judges. This relationship is depicted in Fig. 6 for the POLS and the Eurobarometer items, to give an impression of what the difference between the two methods means for the positions of the boundaries on the reconfigured scales.

From Fig. 6 it can be seen that according to the Semantic Judgement of Word Value in Context Method the interval for the response option “not very satisfied” for the POLS item, does not overlap with the interval for this option as assessed for the similar Eurobarometer item. The latter interval is fully covered by the interval for the response option “fairly satisfied” in the POLS item according to the Semantic Judgement of Word Value in Context Method. When the boundaries are derived from a reference distribution as done in the Reference Distribution Method, they show a dramatic change compared to those obtained using the Semantic Judgement of Word Value in Context Method. The boundaries based on the Reference Distribution Method for the POLS scale are more in harmony with those for the Eurobarometer scale compared to the results obtained using the Semantic Judgement of Word Value in Context Method. Using the Reference Distribution Method, the interval for the response option “very satisfied” of the Eurobarometer scale almost coincides with the combination of the intervals for the response options “very satisfied” and “extra ordinarily satisfied”. On the other side of both scales a similar correlation can be noticed for the interval for the response option “not very satisfied” of the POLS scale with the combined intervals for the response options “not at all satisfied” and “not very satisfied” of the Eurobarometer scale.

### Scale Transformation Using the Reference Distribution Method

In this method the reference distribution used is the beta distribution that fits best to the frequency distribution in a certain year, the reference year, of a happiness item from a deliberately selected survey. Suppose we want to transform the results of another survey for a specific item with a verbal response scale to a continuum from 0 to 10 using the Reference Distribution Method. To do so, given that the results of this other survey are also measured in the reference year, the positions on the continuum from 0 to 10 of the boundaries between the response options of the specific item can be derived from the reference distribution in the way we illustrated in Fig. 5. Once these boundaries have been derived they are kept fixed in the Reference Distribution Method for the transformation of the survey results for the specific item measured in other years. In other words, to transform survey results for other years, the boundaries remain equal to those derived from the reference distribution for the reference year.

The transformation for each of the other years in which the survey has been fielded consists of estimating the parameters of the best fitting beta distribution based on the boundaries derived from the reference distribution and on the frequency distribution of the response on the primary verbal scale in the year in progress. The transformed survey mean is subsequently the outcome of the division of $\hat{\alpha }$ by $\hat{\alpha } + \hat{\beta },$ see formula (Eq. 3) in Sect. 4.1, with $\hat{\alpha }$ and $\hat{\beta }$ the estimated parameters of this best fitting beta distribution. The survey results of a whole time series can be transformed in this way.

In a certain year however, the mode of surveying may be changed. If so, it is plausible that this will influence the position of the boundaries between response options. An example of the effect a mode change can have is the Life Situation Survey of the Sociaal Cultureel Planbureau (SCP) in the Netherlands, which in 2004 was changed from face-to-face interviews responding to a questioner to a paper & pencil survey using a questionnaire.^3^ In such a situation, the position of the boundaries has to be reconsidered and presumably determined anew. To derive new boundaries that comply with the new survey mode, the original reference distribution should not be used. Instead the best fitting beta distribution given the boundaries derived from the original reference distribution and the frequency distribution of the survey results in the year prior or equal to that in which the mode was changed should be selected as a new reference distribution. Whether the new reference distribution should be based on the survey results for the year the mode was changed or for the year prior to that, depends on whether there has been a double measurement: in the ideal situation a survey will be fielded in both modes in the year of change to get insight into the effect of the change. In this case the new reference distribution can be based on the survey results for the same year the mode was changed. If unfortunately no double measurement is available, but the survey results show minor changes from year to year, as a proxy the best fitting beta distribution estimated for the year prior to the year the questionnaire mode was changed can be used.

In the same way, two different surveys to measure happiness that partially overlap in the years they have been fielded can be transformed and combined if a reference distribution is available for one of them. This reference distribution does not necessarily have to be based on a different (third) survey, but can also be derived from one of the two surveys of concern. In this case a reference year has to be selected from the time period in which both surveys have been fielded. Next one of the two surveys should be selected to provide the reference distribution. If the item of interest in this survey has a numerical scale, a reference distribution can be estimated straightforwardly just as it is done for the example from the ESS. If however this item has a verbal scale, the boundaries between the response options must be specified first and the Scale Interval Recorder can be used for this purpose. The reference distribution can be estimated using these specified boundaries and the frequency distribution for the item in the reference year. Given the reference distribution, the time series of both surveys can then be transformed in the way we described earlier.

## Application of the Reference Distribution Method

We will now illustrate how the Reference Distribution Method is used by applying it to the items from POLS and the Eurobarometer for survey results obtained in the years from 1993 to 2009. This application consists of a trend analyses in terms of the comparability of the trends in responses to different questions about happiness in one country. In most of the years of this period, the Eurobarometer was fielded in the spring and in autumn. To demonstrate the Reference Distribution Method, we have selected the results for just one measurement per year. If available, we selected the results obtained in spring otherwise we incorporated the results for autumn.

**Fig. 7:**
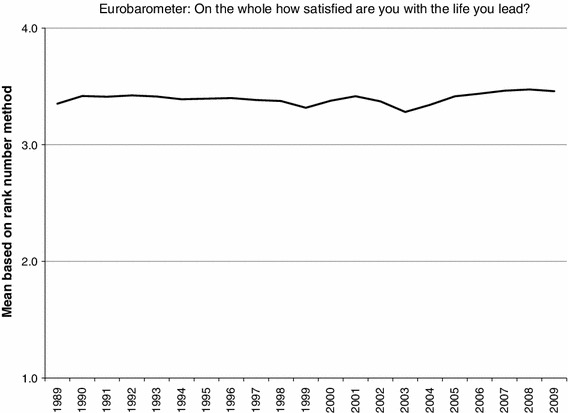
Means of the Eurobarometer item in the period 1989–2009 based on rank numbers primary scale

The means of the Eurobarometer item in the period 1989–2009 when the common Rank Number Method was applied are given in Fig. 7.

In most of the years until 1996 the mean value of the Eurobarometer item was nearly 3.40. In the following years dips were seen in the years 1999 and 2003 and from 2004 the line has climbed to around 3.46 in 2007 and this has been maintained until 2009.

**Fig. 8:**
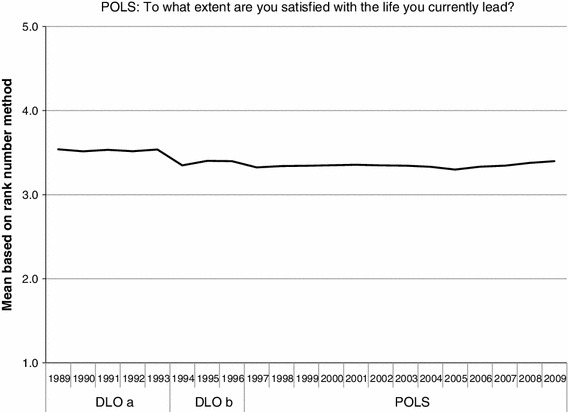
Means of the POLS item in the period 1989–2009 based on rank numbers primary scale

In the period 1989–2009, there were two changes in the POLS survey that affected the responses. The first change was made in 1994 and consisted primarily of a comprehensive revision of the questionnaire forms and a reduction of the survey items in several domains. A major change of the survey design of POLS took place in 1997. Amongst others, the mode of questioning was changed from paper & pencil surveying to face-to-face interviews and instead of drawing samples based on addresses, from then on the sample was drawn based on individual citizens. This change affected the survey results. The name POLS was not used before 1997. In the period from 1989 to 1997 the name of the survey was Doorlopend Onderzoek Leefsituatie, abbreviated to DLO. We present the means of the POLS item for the period 1989–2009, when the common Rank Number Method was used, in Fig. 8. Note, for the years before 1997 we use the abbreviation DLO.

The changes in the design in 1994 and 1997 of POLS are clearly visible in the mean value presented in Fig. 8. In each of the three periods that can be distinguished for the POLS item, the mean values show a rather stable pattern.

We estimated a best fitting beta distribution for the ESS results of 2008 to serve as a reference distribution. We used this reference distribution to derive the boundaries between the response options of the items from both the Eurobarometer and POLS. Using these tuned boundaries we estimated the parameters of the best fitting beta distributions for the POLS results over the years 1997–2009 and for the Eurobarometer results over the years 1994–2009. Fortunately in 1997 the POLS survey was fielded in both the old and the new design, therefore a best fitting beta distribution was available based on the survey results for 1997 according to the new design and on the boundaries derived from the ESS reference distribution. This best fitting beta distribution for 1997 and the survey results over 1997 according to the old design, we used to derive the boundaries between the response options for the survey results obtained in the years 1994–1996. In 1993 there was no double measurement. Therefore we used the beta distribution estimated for 1994 as a reference to transform the survey results obtained in the period 1989–1993.

The time-invariant boundaries as assessed by the judges in the Semantic Judgement of Word Value in Context Method, the boundaries derived from the reference distribution based on the ESS results for 2008 and the adjusted boundaries for the changes in design for the POLS survey in 1997 and 1994 are given in Table 5.

**Table 5:** Upper boundaries of response options for the POLS scale and the Eurobarometer scale

Item code survey	Response options	Upper boundaries			
		Judges	Ref ESS 2008	Ref POLS 1997	Ref POLS 1994
O-SLL-c-sq-v-5-d POLS	Extraordinarily satisfied	10.0	10.0	10.0	10.0
	Very satisfied	8.8	9.0	8.8	8.6
	Satisfied	7.2	7.8	7.5	7.2
	Fairly satisfied	5.3	5.7	5.8	5.5
	Not very satisfied	3.6	4.8	4.9	4.5
O-SLL-u-sq-v-4-b Eurobarometer	Very satisfied	10.0	10.0		
	Fairly satisfied	7.9	7.5		
	Not very satisfied	5.3	4.7		
	Not at all satisfied	3.0	3.6		

In addition to what we exemplified for the difference in the position of the boundaries as presented in Fig. 5 when comparing the Semantic Judgement of Word Value in Context Method and the Reference Distribution Method, we can remark that before the design change of POLS in 1997 the boundaries of the response options in the higher part of the scale were positioned a little lower and those in the lower part of the scale slightly higher. All the boundaries for the period 1989–1993 tuned to the reference distribution for 1994 are positioned somewhat lower on the continuum compared to the boundaries for the period 1994–1996.

**Fig. 9:**
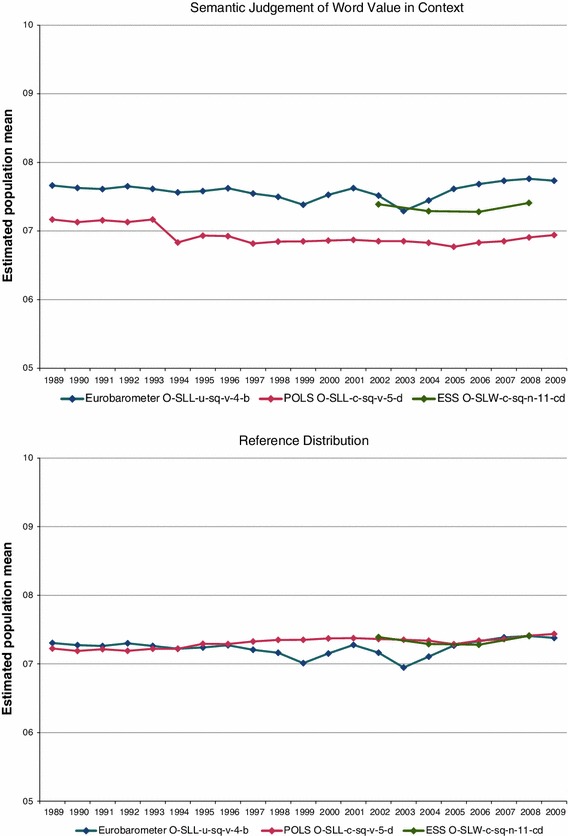
Comparison of the transformation by the Semantic Judgement of Word Value in Context Method and the Reference Distribution Method

In the upper part of Fig. 9 the transformation results according to the Semantic Judgement of Word Value in Context Method are shown and in the lower part the transformation results according to the Reference Distribution Method: for reasons of comparison, besides the transformation results for the POLS and the Eurobarometer items, we have also included in both graphs the transformation results for the ESS item of the survey waves for 2002, 2004, 2006 and 2008.

As can be seen, when applying the Semantic Judgement of Word Value in Context Method, the estimated population means for the Eurobarometer item are too high compared to those for the ESS item, whereas for the POLS item they are too low. The means for POLS when using the Semantic Judgement of Word Value in Context Method furthermore show a large discontinuity in the transition from 1993 to 1994 and a little discontinuity in the transition from 1996 to 1997, which is due to changes in the survey design. After application of the Reference Distribution Method, the transformed survey means for the Eurobarometer item are somewhat lower compared to the application of the Semantic Judgement of Word Value in Context Method, whereas the Reference Distribution Method causes an upward shift for the POLS results. Due to the adjustment of the boundaries for 1993 and 1997, the discontinuities from 1993 to 1994 and from 1996 to 1997 have also disappeared. The fluctuations in each survey over the years turn out to be similar for the results when applying the Reference Distribution Method and the results obtained by the Semantic Judgement of Word Value in Context Method. Application of the Reference Distribution Method brought the results for all three the surveys to a comparable level.

## Discussion

In this paper we gave an overview of the progress made through time in improving methods used to transform ratings on the verbal response scales commonly used in the social sciences to a common numerical or continuous scale, typically ranging from 0 to 10. We ended this overview with a description of the Reference Distribution Method, which in our opinion, provides a valid way to transform ratings on verbal and discrete numerical scales into truly comparable levels on a continuum from 0 to 10.

### Strengths of Scale Homogenization Using a Reference Distribution

The Reference Distribution Method is a variation of the Semantic Judgement of Word Value in Context Method and tunes survey results to the level of a reference distribution in a reference year. We have shown that this Reference Distribution Method is an effective tool for transforming survey results obtained with different items on the same topic to a comparable scale. In addition, the Reference Distribution Method allows corrections to be made for discontinuities due to changes in the design of a survey. As such the Reference Distribution Method can be used to extend time series as it permits combining results from different surveys that have been fielded in, partly, overlapping periods in time.

### Limitations

The Reference Distribution Method can be used to correct much of the differences seen in different sets of findings on happiness that are due to dissimilarity in the measures used; yet it cannot solve all the comparability problems.

One limitation is that the method requires a reference distribution, typically a survey in which the same subject is assessed using a 0–10 numerical scale in the same country in the same year. If not, as a second best option for transforming distributions on numerical scales the Semantic Judgement of Word Value in Context Method should be used, preceded, in the case of a verbal response scale, by a Scale Interval Study.

If a survey has been fielded only once and there is a reference distribution available, then the transformed mean according to the Reference Distribution Method is, by definition, equal to the mean of this reference distribution. This saddles the transformed scores with the errors of the reference distribution, which causes them to become systematic rather than random.

The boundaries between response options that have been derived from a reference distribution are kept fixed as long as the survey design has not undergone a significant change. An obvious question that can be raised is whether it is a reasonable assumption that the boundaries will be more or less fixed over time. The answer is yes, but this will be discussed in an upcoming paper.

The primary verbal scales of the two items we used in this paper to illustrate how the Reference Distribution Method works both had more than three response options. When there are fewer than three i.e. two, response options for a verbal scale the Reference Distribution Method is invalid. There is always a perfectly fitting beta distribution, though with zero degrees of freedom, for a primary scale with only three response options. Some 15 % of the survey studies on happiness in nations is based on 2- and 3-step response scales (Veenhoven 2012) and thus cannot be used for comparison with the other 85 % of the research findings using the Reference Distribution Method.

Another limitation is that the Reference Distribution Method applies only to the diversity in rating scales, that is to the last three aspects of the differences in survey questions presented in Table 1. Survey questions on happiness also differ in the wording of the lead sentence, such as in the key word used, for example ‘happiness’ or ‘satisfaction with life’. Furthermore, the questions differ also in the time frame that is addressed, some referring to ‘current’ happiness, while other ask the respondent to appraise ‘the last year’. In addition to the single questions used here, there are also multiple question inventories, such as Diener et al.’s (1985) five item ‘satisfaction with life scale’. Though each of these items can be tuned in principle, the chance of finding good reference items is lower than for the case of single items.

### Issues for Further Research

Both the Semantic Judgement of Word Value in Context Method and the Reference Distribution Method offer a wide scope of topics for further research.

Results from both the Semantic Judgement of Word Value in Context Method and the Reference Distribution Method are necessary to study the differences between countries in the interpretation of scales and how respondents in practice cope with response options.

The Reference Distribution Method opens the way to combine time series on specific topics taken from different surveys. This is helpful to extend time series, and it will contribute to the development of time series that are more stable over time as the measurements taken from surveys can be averaged for one and the same year.

Finally, in several surveys both happiness and satisfaction with life are assessed, but only in a few cases are they assessed using similar items. This makes it hard to compare the outcomes for both topics. Using the Reference Distribution Method makes it now possible to study whether or not happiness and satisfaction with life constitute basically the same concept and whether or not this is true for all countries or not as the Reference Distribution Method allows us to bring survey data from various sources to a comparable level.

## Conclusion

Survey studies on the same topic often use different questions. One of the differences is in the response scales, which commonly differ in the number of options in verbal and numerical scales used and in the words used to label the response options or scale extremes. As a result much of the available research findings cannot be compared. Several methods have been proposed for transforming observed scores on these different scales into common scores, typically on a 0–10 numerical scale. All of these methods have limitations and the transformed scores they produce appear to differ substantially from distributions obtained directly using 0–10 numerical scales. The Reference Distribution Method proposed in this paper performs better.

## Appendix: Calculation of the Sample Mean After Scale Transformation

### Linear Stretch

In the first step of the Linear Stretch Method the discrete response options of a primary scale are consecutively numbered from p_1_ to p_n_ with n the number of response options. In the next step, each of these numbered options is projected onto a common secondary numerical scale, ranging from a lower bound s_1_ to an upper bound s_n_, such that the option numbered p_1_ is projected to s_1_, the option numbered p_n_ to s_n_ and all other options equally distanced in between. In formula form this can be denoted as:

4 $$ s_{i} = s_{1} + \left( {\frac{{s_{n} - s_{1} }}{{p_{n} - p_{1} }}} \right) \cdot (p_{i} - p_{1} ),\quad i \in \{ 1, \ldots , n\} $$

The sample mean $\bar{x}$ after Linear Stretch, for measured frequencies f_i_, can be calculated as:

5 $$ \bar{x} = s_{1} + \frac{{(s_{n} - s_{1} )}}{{(p_{n} - p_{1} )}} \cdot \left( {\mathop \sum \limits_{i = 1}^{n} (p_{i} - p_{1} ) \cdot f_{i} } \right),\quad i \in \{ 1, \ldots ,n\} $$

For response on a primary scale numbered from 1 to n by steps of 1 and Linear Stretch to scale from 0 to 10, Eq. (5) can be written as:

6 $$ \bar{x} = \frac{10}{(n - 1)} \cdot \left( {\mathop \sum \limits_{i = 1}^{n} i \cdot f_{i} } \right),\quad i \in \{ 1, \ldots , n\} $$

### Semantic Judgement of Fixed Word Value

In the method of Semantic Judgement of Fixed Word Value Method the transformed response options *s*_*1*_ to *s*_*n*_ on the secondary scale are in general, and in contrast to the Linear Stretch Method not equidistance, the formula for calculating the transformed sample mean $\bar{x}$ in Eq. (7) looks slightly different from that in Eq. (6).

7 $$ \bar{x} = \mathop \sum \limits_{i = 1}^{n} s_{i} \cdot f_{i} , \quad i \in \{ 1, \ldots ,n\} $$

## Footnotes

1 The term item is used in this paper to denote the combination of a survey question and its corresponding response options.

2 Permanent Survey on Living Conditions.

3 In the Netherlands in 2004 the Sociaal Cultureel Planbureau (SCP), changed the mode of its Life Situation Survey, from face-to-face interviews using questioners to a paper & pencil questionnaire. This caused a dramatic fall in the percentage of people who rated themselves as either ‘happy’ or ‘very happy’, a drop of 6 percentage points from 2002 to 2004 in a time series that had been rather stable since 1997. This change may be attributed to those responding to a paper & pencil survey having more time to think about their answer than someone doing a face-to-face interview, where the pace is set by the interviewer. This leads to a more cognitive than intuitive response which comes with lower reported happiness (Studer and Winkelmann 2012). Another explanation for the change from 2002 to 2004 is the commonly recognized interviewer bias, caused by the effect an interviewer can have on the response in a face-to-face interview (Katz 1942; Davis et al. 2010). The interviewer may, unintentionally, influence respondents to give for example socially desirable answers that may be more positive than when that respondent answers questions in an uninfluenced situation like a paper & pencil survey.
